# A Dual Binding Mode for RhoGTPases in Plexin Signalling

**DOI:** 10.1371/journal.pbio.1001134

**Published:** 2011-08-30

**Authors:** Christian H. Bell, A. Radu Aricescu, E. Yvonne Jones, Christian Siebold

**Affiliations:** Division of Structural Biology, Wellcome Trust Centre for Human Genetics, University of Oxford, Oxford, United Kingdom; Brandeis University, United States of America

## Abstract

Plexins are cell surface receptors for the semaphorin family of cell guidance cues. The cytoplasmic region comprises a Ras GTPase-activating protein (GAP) domain and a RhoGTPase binding domain. Concomitant binding of extracellular semaphorin and intracellular RhoGTPase triggers GAP activity and signal transduction. The mechanism of this intricate regulation remains elusive. We present two crystal structures of the human Plexin-B1 cytoplasmic region in complex with a constitutively active RhoGTPase, Rac1. The structure of truncated Plexin-B1-Rac1 complex provides no mechanism for coupling RhoGTPase and Ras binding sites. On inclusion of the juxtamembrane helix, a trimeric structure of Plexin-B1-Rac1 complexes is stabilised by a second, novel, RhoGTPase binding site adjacent to the Ras site. Site-directed mutagenesis combined with cellular and biophysical assays demonstrate that this new binding site is essential for signalling. Our findings are consistent with a model in which extracellular and intracellular plexin clustering events combine into a single signalling output.

## Abstract 2

A novel binding site for RhoGTPases on the intracellular region of plexins induces a trimeric ligand—receptor arrangement that appears crucial for plexin function.

## Author Summary

Axon guidance is fundamental to the development of the central nervous system. The growing axon is guided to its correct location by a plethora of extracellular signals. One of the most important extracellular signals is semaphorin, which binds to plexin receptors on the axon. Usually, this kind of extracellular ligand binding is sufficient to transmit the extracellular signal to the intracellular space to trigger changes in the cell, like axon growth. However, activation of plexin receptors requires a “dual” ligand binding: semaphorin on the extracellular side, and a RhoGTPase on the intracellular side. Signal transduction can only occur if both ligands are present. How this intricate regulation mechanism is organized and how concomitant ligand binding can be integrated into a single signalling output within the cell has remained largely unclear. Here, we present crystal structures of one plexin receptor, Plexin-B1, in complex with an intracellular RhoGTPase ligand (Rac1) and show that binding of Rac1 brings together three Plexin-B1 molecules. In this trimeric arrangement each plexin molecule interacts with two Rac1 ligand molecules. This leads to a previously unidentified plexin-Rac1 ligand interface that is crucial for its function. Further biophysical and cellular analysis in combination with previous findings on the extracellular plexin-semaphorin complex allow us to propose a model for how ligand-induced clustering events on the extra- as well as intracellular side are combined to trigger signal transduction.

## Introduction

Plexins constitute a large family of semaphorin receptors that mediate the repulsive chemotactic response necessary for axon guidance in the developing nervous system. They also play an important role in regulating cell migration, angiogenesis, and immune responses [1],[2]. Mutations in plexin receptors have been found in cancers from a variety of tissues [3],[4].

There are four classes of Plexins (A, B, C, and D) [1]. Their architecture is conserved across the family with a large extracellular region including the ligand binding sema domain, a single transmembrane spanning helix, and an intracellular region that transduces signals to a number of downstream pathways [1],[2],[5]. Recently, truncated ectodomain structures of plexins from different classes in complex with their cognate semaphorin ligands have been solved [6]–[8]. They revealed a common architecture in which two plexin monomers bind one semaphorin dimer. This bivalency has been shown to be crucial for the function of the plexin-semaphorin complex [6].

Plexins are transmembrane receptors distinguished by their ability to interact directly with small GTPases of the Ras and Rho family through their intracellular region [9],[10]. They consist of two domains, the GTPase activating protein (GAP) domain, first identified by sequence similarity to RasGAP, and the RhoGTPase binding domain (RBD) [11]–[13]. Recent structural studies of the intracellular region of human Plexin-B1 and mouse Plexin-A3 revealed that the GAP domain is an integral structural unit, with the RBD forming a domain insertion into one of the exposed GAP domain loops [14],. Importantly, the catalytic machinery remained identical, with catalytic arginines found in the same positions in RasGAP and both Plexin-B1 and Plexin-A3 [14]–[17].

Within the plexin family, the human Plexin-B1 signalling pathway is the most extensively characterized to date; two members of the Ras superfamily have been identified as targets of the Plexin GAP activity so far, R-Ras and M-Ras [9],[18]. Inactivation of R-Ras by Plexin-B1 GAP leads to suppression of integrin activation and cell migration, ultimately leading to repulsive axonal guidance [19],[20]. Downregulation of M-Ras leads to reduced dendritic outgrowth and branching [18]. The Plexin-B1 RBD has been shown to bind to the Rho GTPases Rnd1, Rac1, and RhoD exclusively in their active, GTP-bound form [21]–[23]. Small GTPases of the Rho family are key players in remodelling of the actin cytoskeleton and are involved in a plethora of processes initiated by extracellular stimuli [24],[25]. Both Rac1 and Rnd1 are important for the ligand-induced activation of the plexin GAP activity and Rac1 has been found to increase semaphorin binding to Plexin-B1 [19],[26]–[28].

Simultaneous binding of semaphorin on the extracellular side and a RhoGTPase on the intracellular side is a prerequisite for plexin GAP activity [27],[29]. Bivalent semaphorin binding can be mimicked by extracellular, antibody-induced, clustering of the intracellular domain and activation is observed in the presence but not in the absence of Rnd1 [9],[29]. This suggests that semaphorins have a crucial role in bringing together plexin receptors as a step towards activation. Despite a number of structural studies on the plexin RBD and its complex with Rnd1 [15],[30],[31] it remains unclear how RhoGTPases modulate plexins and how the concomitant binding of ligands on the extracellular and the intracellular side of the receptor is integrated into a single signalling output, inactivation of Ras. To address this question we characterized the complex between the intracellular region of Plexin-B1 and a constitutively active form of the RhoGTPase Rac1 both structurally and functionally.

## Results

### Structure of the Intracellular Domain of Plexin-B1 in Complex with Constitutively Active Rac1

**Figure 1:**
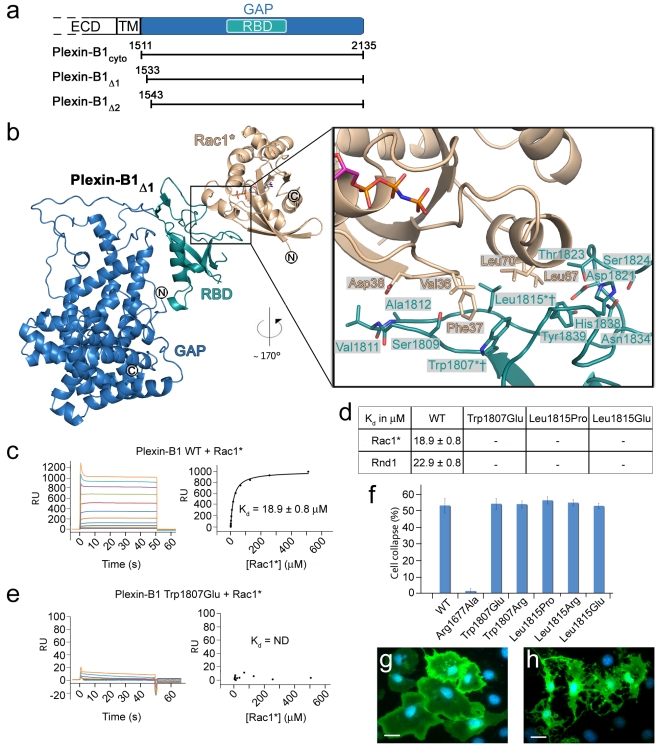
Structure of the monomeric Plexin-B1_Δ1_-Rac1* complex.

Several constructs of the intracellular domain of human Plexin-B1 were designed, of which three, Plexin-B1_cyto_, Plexin-B1_Δ1_, and Plexin-B1_Δ2_, could be solubly expressed in insect cells (Figure 1a). Rac1 was rendered constitutively active by introducing a Gln61Leu mutation [32] in addition to loading with the non-hydrolyzable GTP analogue GppNHp. This Rac1 mutant, expressed in *E. coli*, was used in all subsequent experiments and is named Rac1* hereafter. We have determined the crystal structure of Plexin-B1_Δ1_ in complex with Rac1* to a resolution of 3.2 Å and refined it to a crystallographic R-factor of 20.7% (R_free_ = 23.8%, Figure 1b, Table 1, Figure S1).

**Table 1:** Data collection and refinement statistics.

Data Collection and Refinement	Plexin-B1_Δ1_ – Rac1* Complex	PlexinB1_cyto_ – Rac1* Complex
**Data collection**		
Resolution (Å)	3.20 (3.31–3.20)^a^	4.40 (4.56–4.40)
Space group	C2	I2_1_2_1_2_1_
Cell dimension		
a, b, c (Å)	184.1, 63.9, 84.6	142.1, 224.1, 258.3
α, β, γ (°)	90, 107.5, 90	90, 90, 90
Redundancy	5.2 (5.3)	5.1 (5.2)
Completeness (%)	100 (100)	95.7 (97.5)
R_sym_ (%)	14.3 (65.0)	8.4 (54.4)
Avg I/σ	11.3 (2.5)	15.7 (2.1)
**Refinement**		
Resolution (Å)	46.0–3.2	42.0–4.4
No. Reflections	15,648 (2,807)	25,522 (2,662)
*R*_work_/*R*_free_^b^ (%)	20.7 (25.3)/23.8 (29.6)	23.4 (25.1)/26.4 (26.7)
Complexes in the asymmetric unit	1	3
No. Atoms		
Protein	5,762	16,493
B-factors		
Protein (Å^2^)	66.6	124.6
r.m.s. Deviations		
Bond lengths (Å)	0.008	0.009
Bond angles (°)	0.93	1.16

a Numbers in parenthesis are for the highest resolution shell.

b R_free_ equals the R_work_ against 5% of the data removed prior to refinement.

The overall structures of Plexin-B1_Δ1_ and Rac1* in the complex are very similar to their apo-structures [15],[33] with rmsd values of 1.5 Å and 0.6 Å, respectively. However, there is some flexibility between the Plexin-B1 GAP and the RBD with the RBD being rotated by ∼6° compared to the apo-structure (Figure S2). Rac1* binds exclusively to the RBD and does not form any contacts with the GAP domain. The interface between Rac1* and the Plexin-B1 RBD covers a buried surface area of 707 Å^2^ and is dominated by hydrophobic interactions. Plexin-B1 residues Trp1807^Plex^, Leu1815^Plex^, Thr1823^Plex^, and Tyr1839^Plex^ form a continuous hydrophobic patch that is complemented by Rac1 residues Phe37^Rac^, Val36^Rac^, Leu67^Rac^, and Leu70^Rac^ (Figure 1b). All of these residues are almost completely buried within the interface (at least 80% of the solvent accessible surface area) with the exception of Val36^Rac^ (38%). Thr1823^Plex^ and Tyr1839^Plex^ are part of a potential hydrogen bonding network involving Asp1821^Plex^, Ser1824^Plex^, Asn1834^Plex^, and His1838^Plex^ that is likely to be crucial for the structural integrity of the domain. The hydrophobic interaction between Plexin-B1_Δ1_ and Rac1* is extended by two potential hydrogen bonds formed between the sidechain of Asp38^Rac^ and the backbone amides of Val1811^Plex^ and Ala1812^Plex^. Remarkably, all of the Plexin-B1 residues described above are conserved across A- and B-class plexins (Figure S3), therefore most likely preserving this mode of recognition.

On Rac1*, all residues mentioned above map onto the switch I or switch II region [11] (Figure S4) whose conformation resembles that of active Rac1 in other Rac1-effector complexes [34]. Since these regions undergo large conformational changes upon GTP binding, this explains why Plexin-B1 is highly specific for active, GTP-bound Rac1 [21]. Recently, the structure of the RBD fragment of Plexin-B1 in complex with the constitutively active RhoGTPase, Rnd1, has been reported [15]. Structural superposition of the RBD-RhoGTPase complexes gives an rmsd of 0.96 Å (Figure S5). Despite a sequence identity of only 32% between Rac1 and Rnd1, the Plexin-B1 RBD-Rnd1 complex interface is very similar to the one described here. All hydrophobic interactions as well as the two potential hydrogen bonds are conserved in both structures.

To corroborate our structural findings we studied the affinity between Plexin-B1_cyto_ and Rac1*, as well as Rnd1, using surface plasmon resonance (SPR). Rnd1 is constitutively active due to its lack of GTPase activity [35]. Plexin-B1_cyto_ binds to Rac1 and Rnd1 with an affinity of 18.9 µM and 22.9 µM, respectively (Figure 1c–e, Figure S6), which is in agreement with recently published affinities determined by isothermal titration calorimetry [15]. We found that a series of Plexin-B1 mutations in the hydrophobic interface, Trp1807Glu^Plex^, Leu1815Pro^Plex^ (previously linked to prostate cancer [4]), and Leu1815Glu^Plex^, completely abolished its interactions with Rac1* and Rnd1 (Figure 1d–e, Figure S6).

To validate these effects on binding in a functional context, we performed COS cell-based collapse assays with the full-length transmembrane receptor, testing for Plexin-B1 activity in vivo [36]. Surprisingly, none of the mutants shown to abolish Rac1* or Rnd1 binding had an effect on the collapse response of the cells (Figure 1f–h). We explored this finding further in an independent experimental assay to monitor directly Ras GTPase activity in vivo. In agreement with our results from the collapse assay, none of the interface mutants had an effect on the GAP activity of Plexin-B1 towards R-Ras in this COS cell-based pull-down (Figure S7). Since the necessity of RhoGTPase binding for plexin function is well established [9],[27], it was unclear how to correlate the biophysical and cellular results.

### Three-Fold Arrangement of the Plexin-B1-Rac1 Unit

**Figure 2:**
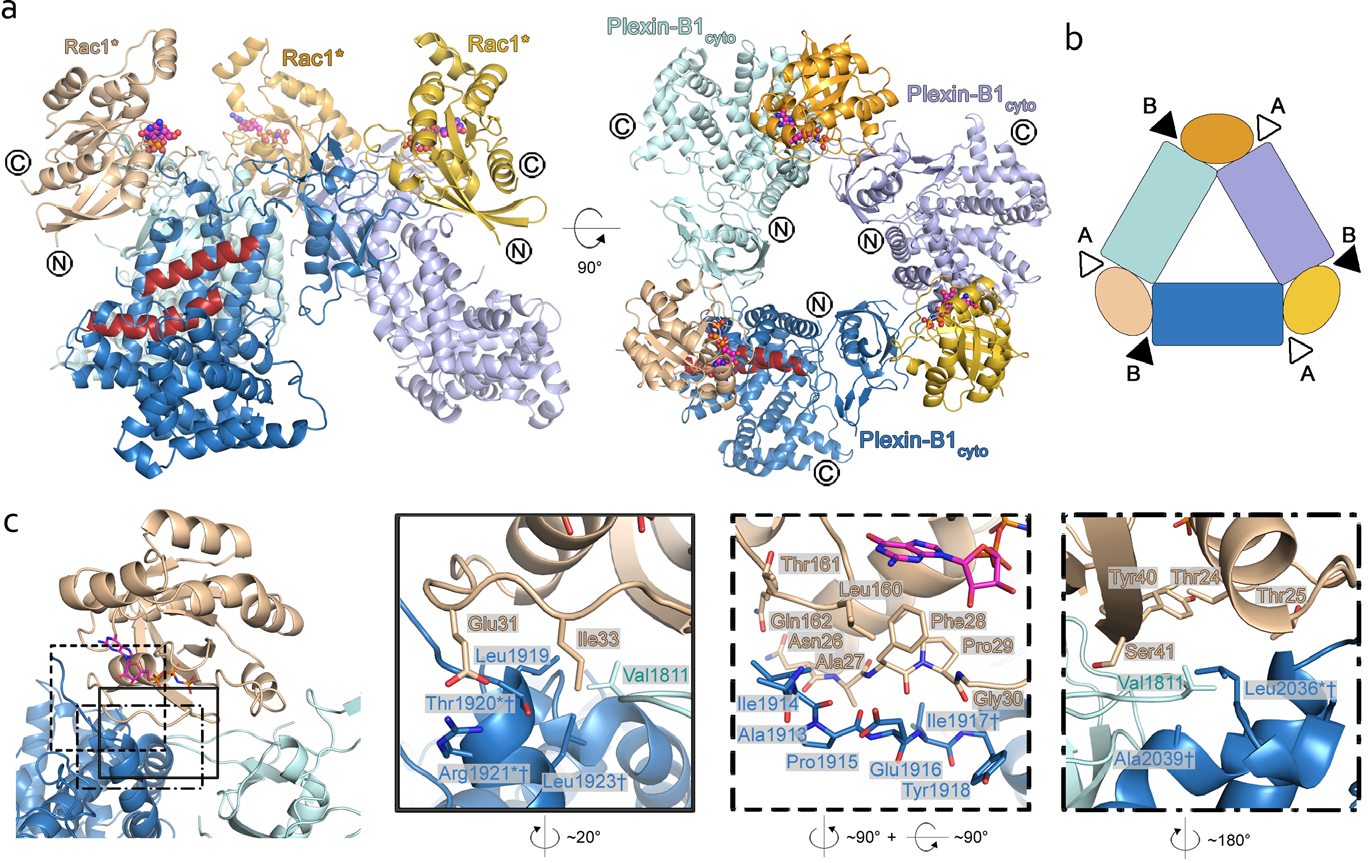
Structure of the 3-fold Plexin-B1_cyto_-Rac1 complex. (a) Overview of the 3-fold arrangement of the three Plexin-B1_cyto_-Rac1* units. Rac1* is coloured in wheat, orange, and yellow, and Plexin-B1_cyto_ is coloured in skyblue, paleblue, and palecyan, respectively. GppNHp is shown as spheres. Termini for Rac1* are labelled in the left panel, and termini for Plexin-B1_cyto_ in the right panel. α-helices 13, 14, and 15 harbouring the putative Ras-binding site are highlighted in red. (b) Schematic overview of the complex. The orientation and colour coding is as in (a), right panel. The two Rac1 binding sites, site A and site B, are marked by arrowheads. (c) Detailed view of the Rac1 binding site B. The orientation of the left panel is similar to Figure 1b, right panel. The three boxed panels correspond to the close-up views depicted in the left panel. Colour coding is as in (a).

The relative position of Rac1* in regard to the putative Ras binding site revealed no mechanism for the direct regulation of the catalytic activity of Plexin-B1 by the small RhoGTPase. To address whether the N-terminal residues missing in the Plexin-B1_Δ1_ construct might harbour an important site for RhoGTPase mediated plexin activity, we solved the crystal structure of the entire cytoplasmic domain of Plexin-B1 (Plexin-B1_cyto_) in complex with Rac1* (Figure 2a). The 4.4 Å model is of high-quality for this resolution range, reflected by the crystallographic R-factor of 23.4% (R_free_ = 26.4%, Table 1, Figure S8). The asymmetric unit contains a trimeric arrangement comprising three copies of the Plexin-B1_cyto_-Rac1* unit, with each Rac1* molecule contacting two Plexin-B1_cyto_ molecules (Figure 2a–b). This arrangement is not the result of crystallographic symmetry but does show near perfect 3-fold geometry (120° between pairs of Rac1* molecules and 117°, 119°, and 124° between the copies of Plexin-B1_cyto_). Moreover, the interfaces between Plexin-B1_cyto_ and Rac1* are essentially identical across the three copies in the asymmetric unit and are not found in any of the crystallographic symmetry generated interfaces. These observations strongly suggest that this 3-fold complex is not purely a product of crystal lattice formation. We were not able to show a 3-fold complex with the soluble constructs in analytical ultracentrifugation at a plexin concentration of 250 µM (unpublished data). This suggests that high local concentrations in the crystal or indeed at the plasma membrane are necessary for this arrangement and that the 3-fold interaction might be too weak to be detected in solution [37].

There are no major conformational changes within the three Plexin-B1_cyto_-Rac1* units when compared to the one-to-one complex. This 3-fold arrangement is mediated by a previously unidentified second binding site for the Rho-GTPase on Plexin-B1_cyto_ (site B, the previously observed Plexin-B1 RBD-RhoGTPase interface hereafter called site A). Site B involves the N-terminal region of α-helix 11 and the loop preceding it (residues 1913^Plex^–1923^Plex^) plus α-helix 16 (residues 2036^Plex^–2039^Plex^) and is in close proximity to the putative Ras binding site (Figure 2a,c, Figure S3). It covers a total buried surface area of ∼570 Å^2^, therefore significantly extending the interface for Rac1 binding, and is dominated by hydrophobic interactions. On Plexin-B1_cyto_ the majority of contacts (60% of buried surface area) are made by the loop residues 1913^Plex^–1918^Plex^ (Figure 2c). Interestingly, these residues are disordered in the apo-structures of Plexin-B1 and Plexin-A3. The site B interface on Rac1* is predominantly formed by residues that precede the switch I region (residues 24–33, Figure 2c). The conformation of these residues is known not to depend on the activation state of the RhoGTPase [11]; thus, the specificity of Plexin-B1 for active RhoGTPases appears to result exclusively from interactions with site A. The 3-fold complex is further stabilized by contacts between two adjacent Plexin-B1_cyto_ molecules, on the one side mainly involving a loop comprising residues 1808^Plex^–1813^Plex^, and on the other side a surface directly adjacent to site B (residues 1919^Plex^–1938^Plex^ and residues 2036^Plex^–2044^Plex^, Figure 2c). However, this plexin-plexin interaction is unlikely to be stable without the addition of the bridging Rac1* since it only contributes a total buried surface area of ∼310 Å^2^.

**Figure 3:**
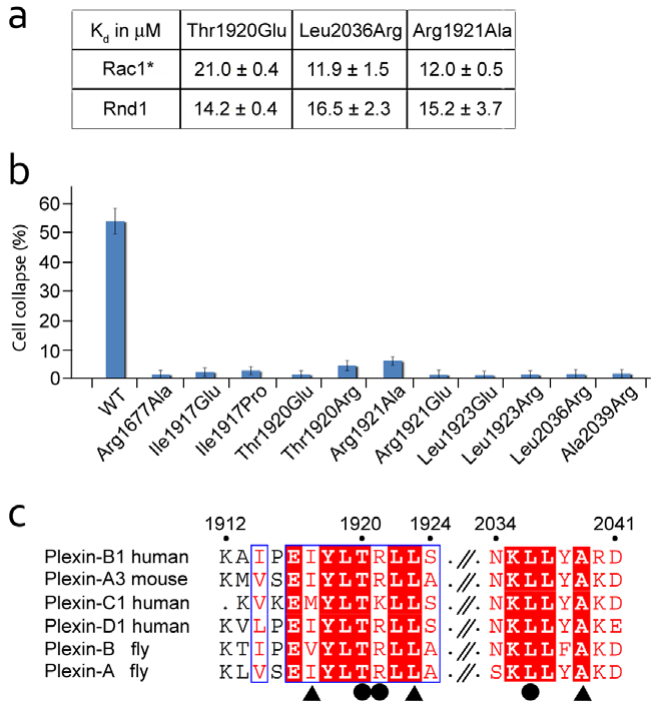
Characterization of the second binding site. (a) Binding constants (*K*_d_) measured by SPR between different Plexin-B1 constructs carrying mutations in binding site B, and Rac1* and Rnd1, respectively. Data are expressed as mean ± standard error. (b) Histogram showing the effect of different site B mutations on semaphorin-induced collapse. Results are shown as mean with error bars representing standard error of the mean. (c) Sequence alignment of residues involved in site B interaction. Residues that were mutated in the cell collapse assay are marked with a triangle and those that were analysed in both SPR and cellular assays are marked with a dot.

In order to assess the potential functional significance of site B, we designed three Plexin-B1 mutants, Thr1920Glu^Plex^, Arg1921Ala^Plex^, and Leu2036Arg^Plex^. We first studied the binding affinity of these mutants to Rac1* and Rnd1 using SPR. None of the site B mutations had a significant effect on the affinity towards either of the RhoGTPases, suggesting that site A alone is sufficient for Rac1* and Rnd1 binding (Figure 3a, Figure S9). However, these mutations as well as additional ones at these and other site B residues (Ile1917^Plex^, Leu1923^Plex^, and Ala2039^Plex^) completely abolished the typical collapse response in the COS-cell assay (Figure 3b). Every site B mutation tested was detrimental to Plexin-B1 activity and led to a complete loss of function. All mutant proteins showed a similar expression level, as judged by immunofluorescence, and were present in the plasma membrane, indicating their structural integrity (unpublished data). In the same background, the Plexin-B1 site B mutants also did not show any GAP activity towards R-Ras, thus further validating the findings of the collapse assay (Figure S7). These results indicate that although site B is not essential for binding of the RhoGTPase, it is crucial for Plexin-B1 activity, suggesting that the 3-fold complex seen in the crystal has functional significance. In accordance with this putative functional role, all of the residues in site B are conserved across all species and classes of plexins with the exception of Ala1913^Plex^ and Pro1915^Plex^, whose sidechains do not participate in the Rac1-site B interaction (Figure 3c).

### The Importance of the N-Terminal Segment of the Plexin-B1 Cytoplasmic Region

**Figure 4:**
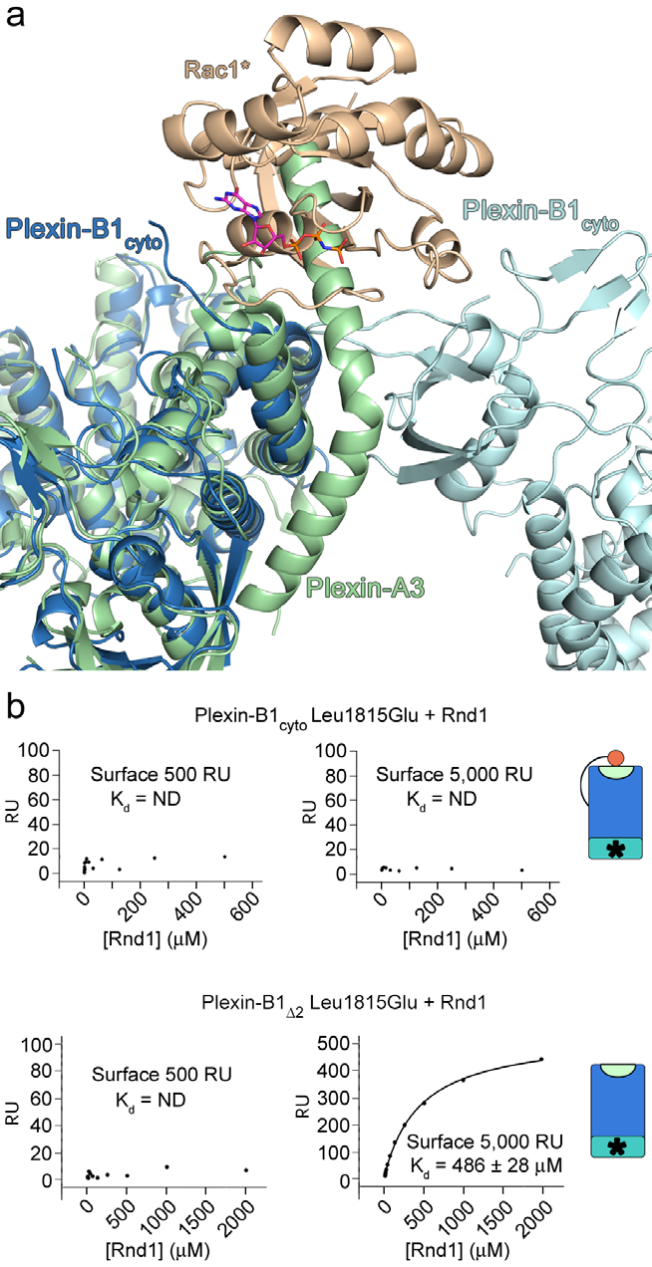
Analysis of the Plexin-B1_cyto_ N-terminal juxtamembrane helix. (a) Superposition of mouse Plexin-A3 (PDB ID: 3IG3) onto the 3-fold Plexin-B1_cyto_-Rac1* complex. Colour coding is as in Figure 2a. Plexin-A3 is shown in pale green. The juxtamembrane helix ordered in the Plexin-A3 structure would block Rac1* binding to site B. (b) Representative plots of the equilibrium binding response against Rho GTPase concentration ranging from 120 nM to 2,000 µM. ND, not determinable. The plexin constructs used in each experiment are schematically presented on the right with colour coding as in Figure 5.

The 3-fold complex revealed by the crystal structure and the cell collapse assays suggest that GTPase binding at site B contributes to plexin function. However, the SPR experiments reveal no direct evidence for GTPase binding at this site. We therefore sought an explanation for this lack of binding. In both the one-to-one and 3-fold complex structures, we were unable to trace the N-terminal helix (residues 1511^Plex^–1562^Plex^, Figure S3) due to a lack of well-ordered electron density. Interestingly, there is a similar absence of electron density in this region for the high resolution apo-structure of Plexin-B1 [15]. This suggests that the N-terminal helix of Plexin-B1 has some internal flexibility, likely around the hinge region adjacent to Ile1563. In agreement with this, the three residues preceding Ile1563 are Gly1562, Ser1561, and Gly1560, which may allow large conformational freedom of the N-terminal helix even in a trimeric arrangement. In contrast, in the apo-structure of mouse Plexin-A3 [14], this region was well-defined. Superposition of the Plexin-A3 structure with the 3-fold complex reveals that this helix would block site B, therefore preventing its interaction with Rac1* (Figure 4a). This steric hindrance model predicts that shortening of the N-terminal helix will remove this block and allow Rac1* and Rnd1 to bind to site B.

To test this model we generated mutant constructs lacking the N-terminal helix (Plexin-B1_Δ2_) and assayed for RhoGTPase binding in SPR. Indeed, RhoGTPase binding to the site A Plexin-B1_Δ2_ mutant Leu1815Glu^Plex^ was now observed, suggesting that truncation of the N-terminal helix has exposed site B (Figure 4b, Figure S10). However, binding can only be observed with high coupling densities of the Plexin-B1_Δ2_ on the SPR chip (Figure 4b). This is consistent with a bivalency effect in which two adjacent plexin molecules bind the same RhoGTPase molecule, implying that the mutated site A is still competent to contribute to an avidity effect [38]. At low coupling densities the Plexin-B1_Δ2_ molecules are too far apart from each other to allow a bivalent interaction with Rac1* or Rnd1. We did not observe an increase in affinity for wild-type N-terminal truncated Plexin-B1_Δ2_ compared to the full-length Plexin-B1_cyto_ even at high coupling densities (unpublished data). Binding studies on the isolated Plexin-B1 RBD show similar affinities [21] to those we determined for the full-length cytoplasmic region. Thus these observations suggest that, if intact, site A dominates binding to the RhoGTPase.

Interestingly, sequence analysis of the N-terminal cytoplasmic segment of Plexin-B1 (residues 1511–1539) predicts a trimeric coiled-coil (Figure S11) and similar regions in other plexins from all classes are also predicted to adopt a coiled-coil conformation. In accordance with this, Ile1563^Plex^ in Plexin-B1_cyto_, the first residue visible in the electron density map, points towards the inside of the 3-fold complex locating the three N-terminal segments in close proximity to each other (Figure 2a, right panel). This proximity suggests an explanation for the observation of the higher oligomeric state 3-fold complex in the crystal. Although the N-terminal helix is not well-ordered in the structure, it could form a trimeric coiled-coil, albeit containing significant flexibility.

## Discussion

Plexin-semaphorin signalling is dependent on signals from both the extra- and intracellular side. Several studies have shown that both semaphorin binding on the outside and RhoGTPase binding on the inside of the cell are required for plexin activity to occur [9],[27]. The nature of these signals and how they are integrated into a single output, namely RasGAP activity, has been a critical question in this field and several models have been proposed [6]–[8],[14],[15],[39].

Recently, several structures of truncated plexin ectodomains in complex with their cognate semaphorins have been reported [6]–[8]. Despite ranging across three different classes, all of these ectodomain complexes share the same overall architecture with one semaphorin dimer bringing together two plexin monomers. In combination with a detailed biophysical and cellular characterization, these structural data have led to the proposal that the bivalency effect is a prerequisite for plexin signalling [6],[8].

For the cytoplasmic region, our structures of Plexin-B1 in complex with Rac1* do not show major structural rearrangements when compared to the apo-structure of Plexin-B1 [15]. For the one-to-one complex, Rac1* is positioned distant from the Ras binding site on the Plexin-B1_Δ1_ molecule. This excludes the possibility of a direct interaction or regulation of RasGAP activity by the RhoGTPase. Instead, the 3-fold complex reveals an additional binding site on a neighbouring Plexin-B1_cyto_ molecule that is in close proximity to the predicted Ras binding site. This interaction may result in a small conformational change in the Ras binding region, although a detailed analysis of these changes cannot be made due to the low resolution of our data. It is, however, noteworthy that allosteric regulation of Ras binding by RhoGTPase binding has been proposed by He et al. based on a homology model of the Plexin-A3-Rnd1 complex [14]. We cannot exclude the possibility that within the protein crystal the trimeric arrangement is favoured over other site B mediated oligomeric states due to the lattice contacts. Indeed, the 3-fold arrangement constitutes the asymmetric unit in the crystal and therefore accommodates slight variations between the three Plexin-B1-Rac1 units (see Results section).

**Figure 5:**
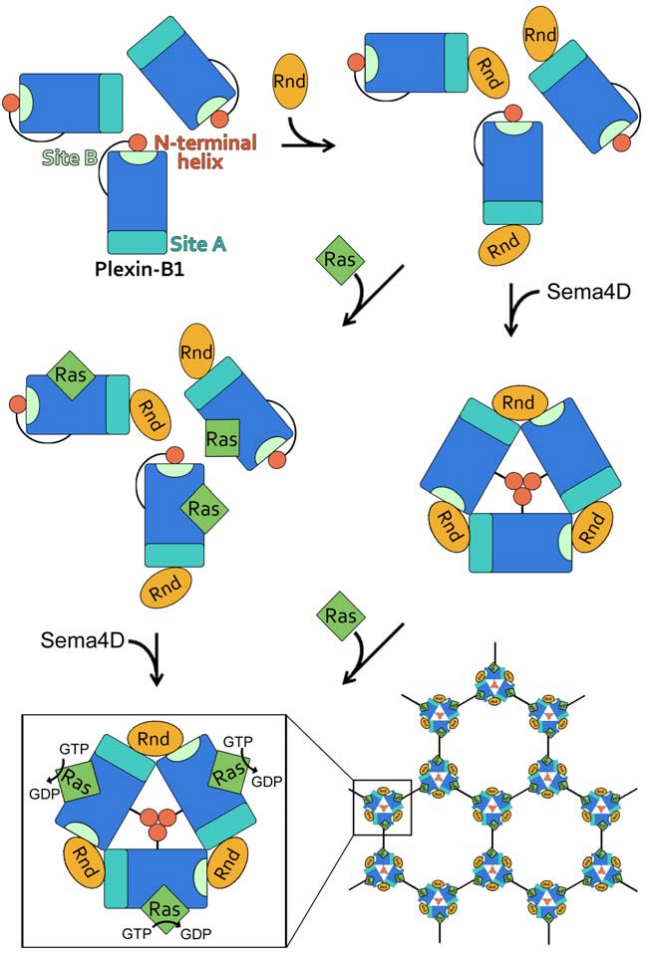
Model for intracellular plexin signalling. We propose that the Ras-GAP activity of Plexin-B1 is a result of a two-step signalling process. Step one would consist of Rnd1 binding to the intracellular Plexin-B1 RBD region. Binding of Ras molecules to this complex cannot result in GTPase activity unless a second binding event, involving extracellular semaphorin-mediated plexin clustering, induces formation of the intracellular 3-fold Plexin-B1-Rnd1 complexes. Such an arrangement is stabilised by the interaction of Rnd1 with a novel binding site on a neighbouring Plexin-B1 molecule, exposed following the displacement of a juxtamembrane helix at the N-terminal of the plexin intracellular region. In this clustered arrangement RasGAP activity can occur and Ras gets inactivated. The trimer on the inside, in conjunction with semaphorin-plexin dimers on the extracellular side, may lead to the formation of an extended signalling array. R-Ras/M-Ras (Ras) and Rnd1 (Rnd) are depicted in their GTP-bound form. Plexin-B1_cyto_ is depicted in blue rectangle, with sites A and B highlighted. The mobile juxtamembrane helix at the N-terminal of the plexin intracellular region is indicated by a red disc. The two GTPases are shown as orange ovals (Rnd1) and green diamonds (Ras), respectively. Semaphorin induced dimerization of the plexin ectodomain is indicated by black lines.

The occurrence of a 3-fold arrangement in crystals of Plexin-B1-Rac1 complexes appears to be dependent on the juxtamembrane, N-terminal helix. Physiologically, this region connects the intracellular domain with the transmembrane and extracellular region. This suggests a mechanism by which both semaphorin binding on the outside and RhoGTPase binding on the inside are connected to result in RasGAP activity (Figure 5). The first step in this model is binding of the RhoGTPase to binding site A of the intracellular domain. Although RhoGTPase binding has been shown to be a prerequisite for Ras binding, it is not sufficient to trigger signalling [9]. Semaphorin binding on the outside of the cell may result in clustering of the receptors [6] either from an autoinhibited, monomeric, or dimeric state [14],[15],[39]. Such extracellular rearrangement could be transmitted to the intracellular N-terminal helix. The rearrangement of this juxtamembrane helix would free up binding site B, allowing the RhoGTPase to bridge two plexin molecules and stabilize the 3-fold arrangement. Formation of a trimeric cluster could result in the proper positioning of the catalytic machinery allowing RasGAP activity to occur, since it has been shown that clustering of the intracellular domain is crucial for this activity [9]. In summary, we propose that receptor clusters nucleated by the dimeric complex on the extracellular side and the trimeric complex on the intracellular side will integrate both RhoGTPase and Semaphorin binding into a single signalling output.

## Materials and Methods

### Protein Expression and Purification

A series of constructs of the intracellular domain of human Plexin-B1 (GenBank ID: NP_001123554) lacking both C- and N-terminal regions as well as the RBD were designed and cloned into pBacPAK9 with a C-terminal His_6_-Tag for purification. Of these constructs three could be solubly expressed via baculovirus infection in Sf9 cells (Plexin-B1_Δ1_, residues 1533–2135; Plexin-B1_Δ2_, residues 1543–2135; and Plexin-B1_cyto_, residues 1511–2135). Cells were harvested at 2,000×g for 15 min, resuspended in binding buffer (20 mM phosphate, pH, 7.4, 500 mM NaCl, 0.5 mM β-mercaptoethanol), sonicated, and then centrifuged at 46,000×g for 1 h at 4°C. The supernatant was collected and the protein was purified by ion metal affinity chromatography followed by size exclusion chromatography in 10 mM Hepes, pH 7.5, 150 mM NaCl, 2 mM TCEP [40].

Mutations were generated by a two-step overlapping PCR using Pyrobest Polymerase (Takara). Mutant plexin constructs used for SPR studies were expressed in human HEK 293T cells essentially as described [41]. Three days after transfection the cells were harvested and purified following the protocol used for the wild-type proteins. All mutant proteins had similar expression level compared to Plexin-B1_cyto_ as determined by SDS-PAGE.

Rac1 Gln61Leu (residues 1–176, GenBank ID: CAB53579) and Rnd1 (residues 5–200, GenBank ID: BAB17851) were cloned into the expression vector pET22b, expressed in *E. coli* BL21 Star (Invitrogen), and purified following an established protocol described elsewhere [40]. After purification Rac1 was incubated with 10 mM EDTA, pH 8.0, and calf intestine alkaline phosphatase (NEB) to degrade any bound nucleotide. Subsequently the protein was loaded with the non-hydrolyzable GTP analogue GppNHp and purified by size exclusion chromatography in 10 mM Hepes, pH 7.5, 150 mM NaCl, 2 mM MgCl_2_, 2 mM TCEP.

SEMA4D_ecto_ (residues 22–677) was expressed in CHO lecR cells as previously described [6].

The Ras binding domain of Raf-1 (residues 51–131) was fused to GST (GST-RBD), expressed in *E. coli* BL21 Star (Invitrogen), and purified following an established protocol described elsewhere [40].

### Crystallization, Data Collection, and Structure Refinement

Prior to crystallization all proteins were concentrated by ultrafiltration to 10 mg/ml and complexes were formed by mixing Plexin-B1 and RhoGTPase in a 1∶1.2 molar ratio. Nano-litre crystallization trials were set-up using a Cartesian Technologies robot (100 nl protein solution plus 100 nl reservoir solution) in 96-well Greiner plates [42], placed in a TAP Homebase storage vault maintained at 295 K, and imaged via a Veeco visualization system [43]. The PlexinB1_cyto_-Rac1* complex crystallized in 1 M Li_2_SO_4_, 0.5 M ammonium sulphate, 0.1 M citrate, pH 5.6, and Plexin-B1_Δ1_-Rac1* complex crystallized in 20% PEG 3350, 0.2 M KSCN, 0.1 M Bis-Tris Propane, pH 6.5.

Diffraction data were collected at 100 K with the crystals being flash-cooled in a cryo N_2_ gas stream. Prior to flash-freezing, crystals were treated with a cryo protectant solution consisting of 25% (v/v) glycerol in mother liquor. The Plexin-B1_Δ1_-Rac1* crystals crystallized as thin needles and data were collected at the microfocus beamline ID23-2 at the European Synchrotron Radiation Facility, France, following a helical data collection strategy. Plexin-B1_cyto_-Rac1* crystals crystallized as thin squares and data were collected at beamline I03 at Diamond Light Source, UK. X-ray data were processed and scaled with the HKL suite [44]. Data collection statistics are shown in Table 1.

Both structures were solved by molecular replacement using PHASER [45] with the structure of human Plexin-B1 (PDB ID: 3HM6 [15]) and active Rac1 (PDB ID: 1MH1 [33]) as search model. The solution was manually adjusted using COOT [46] and refined using autoBUSTER [47]. Refinement statistics are given in Table 1; all data within the indicated resolution range were included. The 4.2 Å structure was refined using 3-fold NCS as implemented in autoBUSTER [47] and tight geometric restraints to minimize the introduction of any model bias.

Stereochemical properties were assessed by MOLPROBITY [48]. Ramachandran statistics are as follows (favoured/disallowed (%)): Plexin-B1_cyto_-Rac1* 91.7/0.2, Plexin-B1_Δ1_-Rac1* 95.5/0.2 (pre-proline residue Leu1981 is in a disallowed region in both structures). Superpositions were calculated using SHP [49]. Buried surface areas of protein-protein interactions were calculated using the PISA webserver (http://www.ebi.ac.uk/msd-srv/prot_int/pistart.html).

### Surface Plasmon Resonance Binding Studies

SPR experiments were performed using a Biacore T100 machine (GE Healthcare) at 25°C in standard buffer supplemented with 0.05% (v/v) Tween 20. Protein concentrations were determined from the absorbance at 280 nm using calculated molar extinction coefficients. All plexin constructs for surface attachment were enzymatically biotinylated within an engineered C-terminal tag. These proteins were then attached to surfaces on which 5,000 RU of streptavidin were coupled via primary amines [50] yielding a density of 500–5,000 response units (RU) of biotinylated protein. All experiments were done in duplicates with independently purified proteins. The signal from experimental flow cells was corrected by subtraction of a blank and reference signal from a mock or irrelevant protein coupled flow cell. In all experiments analyzed, the experimental trace returned to baseline after each injection and the data fitted to a simple 1∶1 Langmuir model of binding. *K*_d_ values were obtained by nonlinear curve fitting of the Langmuir binding isotherm (bound = C^*^max/(*K*_d_+C), where C is analyte concentration and max is the maximum analyte binding) evaluated using the Biacore Evaluation software (GE Healthcare).

### Functional Cell Collapse Assay

Cellular collapse assays were performed essentially as described [36]. Briefly, COS-7 cells were seeded on glass coverslips and transfected with full-length human Plexin B1 carrying an N-terminal Flag-tag essentially as described [42]. Two days after transfection, cells were treated with medium containing secreted SEMA4D_ecto_ and incubated for 30 min at 37°C. Finally, the cells were fixed and stained with anti-Flag primary antibody (Sigma) and Alexa 488-labelled secondary antibody (Invitrogen). Cell nuclei were counterstained with DAPI (Invitrogen) and cells were visualized with a TE2000U fluorescence microscope (Nikon) equipped with an Orca CCD camera (Hamamatsu). Plexin B1-expressing cells were classified as collapsed or non-collapsed on the basis of reduced surface area. Each experiment was repeated twice and 2×200 cells were counted each time. Results are shown as mean with error bars representing standard error of the mean.

### Pull-Down Assay

Pull-down assays were performed essentially as described [51]. COS-7 cells were seeded in 6-well dishes and transfected with full-length human Plexin-B1 and its mutants, respectively, and R-Ras. Two days after transfection, cells were treated with medium containing secreted SEMA4D_ecto_ and incubated for 10 min at 37°C. Cells were washed twice with ice-cold phosphate-buffered saline and then lysed with lysis buffer (50 mM Tris-HCl, pH 7.5, 200 mM NaCl, 5 mM MgCl_2_, 10% glycerol, 1% Non-ident P-40 substitute, 2 mM β-mercaptoethanol). Cell lysates were incubated with GST-RBD pre-coupled to glutathione-agarose beads (GE Healthcare) for 45 min at 4°C. After three wash steps with lysis buffer the beads were collected in Laemmli sample buffer and analyzed by SDS-PAGE and immunoblotting with R-Ras- and GST-specific antibodies, respectively.

#### Accession codes

Atomic coordinates and structure factors of the Plexin-B1_Δ1_-Rac1* and the Plexin-B1_cyto_-Rac1* complexes have been deposited in the Protein Data Bank with accession numbers 3SU8 and 3SUA, respectively.

## Supporting Information

Figure S1 Stereoview of the electron density of the Plexin-B1_Δ1_-Rac1* interface. The orientation is similar to Figure 1b, right panel. The density represents a 3.2 Å SigmaA-weighted 2*Fobs-Fcalc* map contoured at 1.0 σ.
(TIF)

Figure S2 Superposition of the Plexin-B1apo structure onto the Plexin-B1_Δ1_-Rac1* complex. Colour coding is as in Figure 1b with Plexin-B1_apo_ in pale green. Coordinates for the Plexin-B1apo structure can be found under PDB ID: 3HM6. The complexes were aligned onto the plexin GAP domains using SHP [49]. The orientation is similar to Figure 1b, right panel. The slight rotation of the Plexin-B1_Δ1_ RBD in comparison to the Plexin-B1_apo_ structure is indicated by an arrow.
(TIF)

Figure S3 Sequence alignment of the intracellular region of plexins from different classes and organisms. The plexin sequences were aligned using MULTALIN (bioinfo.genotoul.fr/multalin/multalin.html) and formatted with ESPRIPT (espript.ibcp.fr/ESPript/ESPript/). Numbering corresponds to the full-length human Plexin-B1. Secondary structure elements are shown for human Plexin-B1. Residues that were mutated and studied in SPR as well as in the cellular collapse assays are marked with a dot; those that were only studied in the cellular assay are marked with a triangle.
(TIF)

Figure S4 Sequence alignment of the human RhoGTPases Rac1, Rnd1, and RhoD. The alignment is prepared as described in Figure S2. Numbering corresponds to human Rac1. Secondary structure elements are shown for human Rac1. The three regions characteristic for small GTPases and their activation state, the P-loop, switch I, and switch II, are marked by yellow boxes.
(TIF)

Figure S5 Superposition of the Plexin-B1 RBD-Rnd1 complex onto the Plexin-B1_Δ1_-Rac1* structure. Colour coding is as in Figure 1b. Rnd1 is in pale green and the Plexin-B1 RBD of the Plexin-B1 RBD-Rnd1 complex in purple. Coordinates for the Plexin-B1 RBD-Rnd1 complex can be found under PDB ID: 2REX. The complexes were aligned onto the plexin molecules using SHP. The orientation is similar to Figure 1b, right panel, with Rnd1 residues labelled in pale green.
(TIF)

Figure S6 Binding of Rac1* and Rnd1 to site A mutants of Plexin-B1. Left, representative sets of experimental sensorgrams from typical equilibrium-based binding experiments, with reference subtraction. Different concentrations of the respective RhoGTPase were injected over surfaces coupled with the plexin constructs. For all injections, the experimental traces reached equilibrium and returned to baseline after the injection. Right, plot of the equilibrium binding response (response units (RU)) against RhoGTPase concentration ranging from 120 nM to 500 µM. Within one experiment each concentration was measured twice. All experiments were performed in duplicate. Best-fit binding curves corresponding with a 1∶1 binding model are shown as lines. Binding constants (K_d_) are given as mean with the error representing the standard error of the mean. WT, wild-type; ND, not determinable. (a) Plexin-B1_cyto_ Leu1815Pro+Rac1*, (b) Plexin-B1_cyto_ Leu1815Glu+Rac1*, (c) Plexin-B1_cyto_ WT+Rnd1, (d) Plexin-B1_cyto_ Trp1815Glu+Rnd1, (e) Plexin-B1_cyto_ Leu1815Pro+Rnd1, and (f) Plexin-B1_cyto_ Leu1815Glu+Rnd1.
(TIF)

Figure S7 Mutations in site B but not site A abolish Plexin-B1 RasGAP activity. COS-7 cells transfected with full-length Plexin-B1 and its mutants were stimulated with SEMA4D_ecto_ for 10 min. The cell lysates were incubated with GST-fused Ras-binding domain of Raf-1 and bound R-Ras and total cell lysates were detected by immunoblotting. The results shown are representative of two independent experiments that yielded similar results. WT, wild-type; mock, chicken receptor protein tyrosine phosphatase σ Ig1-2.
(TIF)

Figure S8 Stereoview of the electron density of the Plexin-B1_cyto_-Rac1* site B interface. The orientation is similar to Figure 2b, third panel. The density represents a 4.4 Å SigmaA-weighted 2*F_obs_-F_calc_* map contoured at 1.0σ.
(TIF)

Figure S9 Binding of Rac1* and Rnd1 to site B mutants of Plexin-B1. Data are presented as in Figure S5. (a) Plexin-B1_cyto_ Thr1920Glu+Rac1*, (b) Plexin-B1_cyto_ Leu2036Arg+Rac1*, (c) Plexin-B1_cyto_ Arg1921Ala+Rac1*, (d) Plexin-B1_cyto_ Thr1920Glu+Rnd1, (e) Plexin-B1_cyto_ Leu2036Arg+Rnd1, and (f) Plexin-B1_cyto_ Arg1921Ala+Rnd1.
(TIF)

Figure S10 Binding of Rac1* and Rnd1 to Plexin-B1_cyto_ and Plexin-B1_Δ2_. Data are presented as in Figure S5. (a) Plexin-B1_cyto_ Leu1815Glu+Rac1*, 500 RU loaded on the chip, (b) Plexin-B1_cyto_ Leu1815Glu+Rnd1, 500 RU loaded on the chip, (c) Plexin-B1_Δ2_ Leu1815Glu+Rac1*, 500 RU loaded on the chip, (d) Plexin-B1_Δ2_ Leu1815Glu+Rnd1, 500 RU loaded on the chip, (e) Plexin-B1_Δ2_ Leu1815Glu+Rac1*, 5,000 RU loaded on the chip, and (f) Plexin-B1_Δ2_ Leu1815Glu+Rnd1, 5,000 RU loaded on the chip. Data for binding of Rac1* or Rnd1 to Plexin-B1_cyto_ Leu1815Glu with 5,000 RU loaded on the chip can be found in Figure S5b and S5f, respectively.
(TIF)

Figure S11 The juxtamembrane helix is predicted to form a trimeric coiled-coil. Coiled-coil probabilities were calculated for human Plexin-B1 using MultiCoil (http://groups.csail.mit.edu/cb/multicoil/cgi-bin/multicoil.cgi) and plotted against residue number. Overall probabilities are shown in dashed blue, trimeric coiled-coil probabilities in green, and dimeric coiled-coil probabilities in red. The domain organization corresponding to the residue numbers is shown under the graph.
(TIF)
