# Supplementary figures and images for: A Dual Binding Mode for RhoGTPases in Plexin Signalling
Source: PLoS Biol. 2011 Aug 30;9(8):e1001134. doi: 10.1371/journal.pbio.1001134 (PMC3166162; doi:10.1371/journal.pbio.1001134)

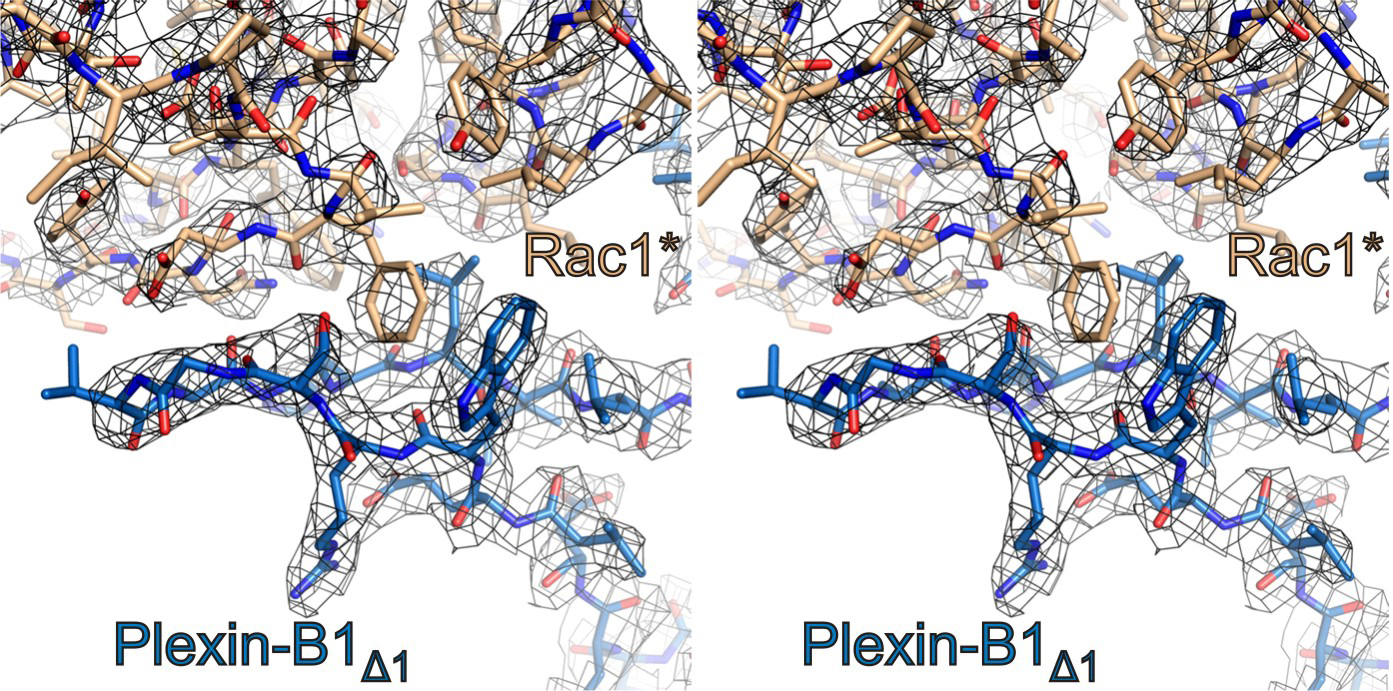

Supplement: Figure S1 — Stereoview of the electron density of the Plexin-B1Δ1-Rac1* interface. The orientation is similar to Figure 1b, right panel. The density represents a 3.2 Å SigmaA-weighted 2Fobs-Fcalc map contoured at 1.0 σ. (TIF) [file pbio.1001134.s001.tif]

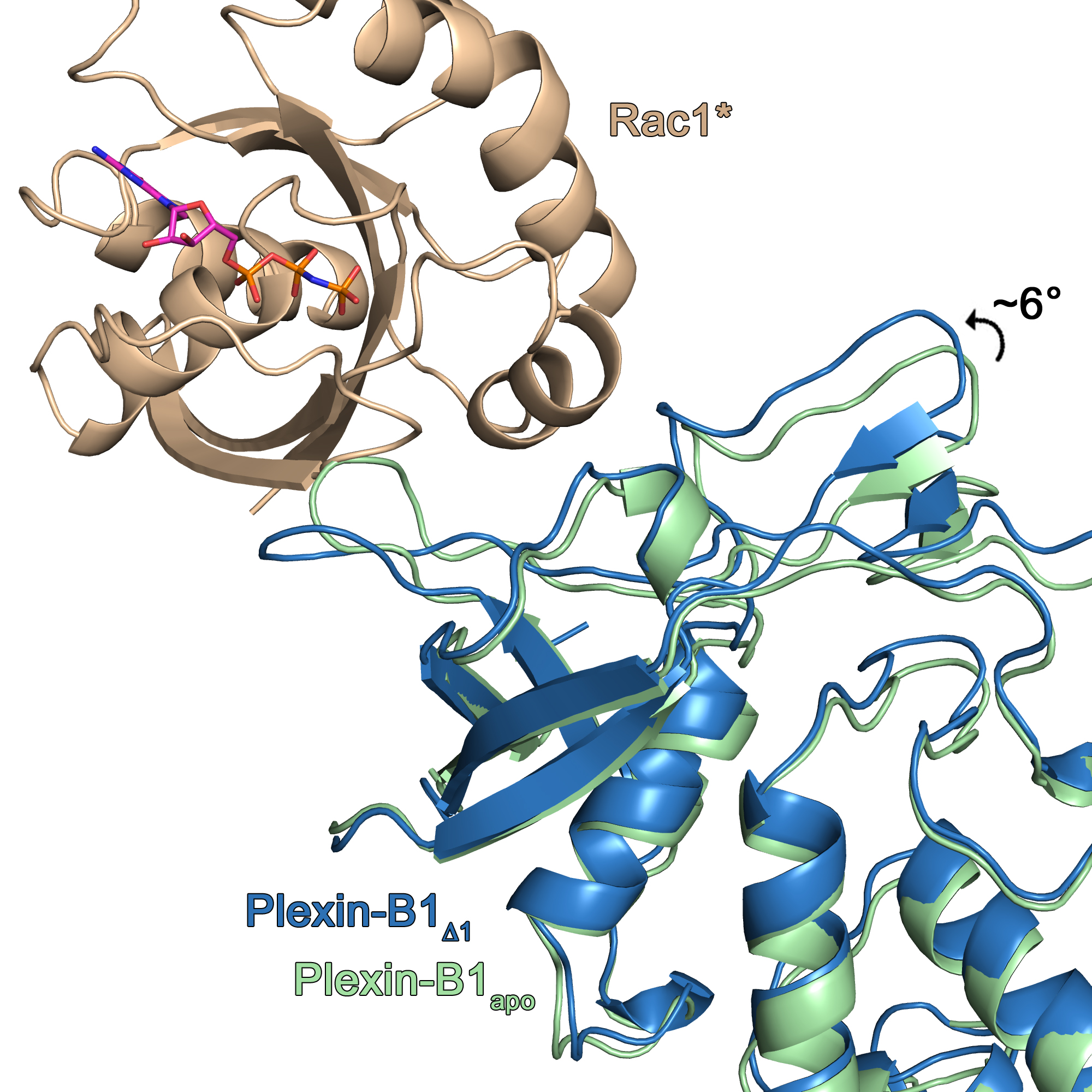

Supplement: Figure S2 — Superposition of the Plexin-B1apo structure onto the Plexin-B1Δ1-Rac1* complex. Colour coding is as in Figure 1b with Plexin-B1apo in pale green. Coordinates for the Plexin-B1apo structure can be found under PDB ID: 3HM6. The complexes were aligned onto the plexin GAP domains using SHP [49]. The orientation is similar to Figure 1b, right panel. The slight rotation of the Plexin-B1Δ1 RBD in comparison to the Plexin-B1apo structure is indicated by an arrow. (TIF) [file pbio.1001134.s002.tif]

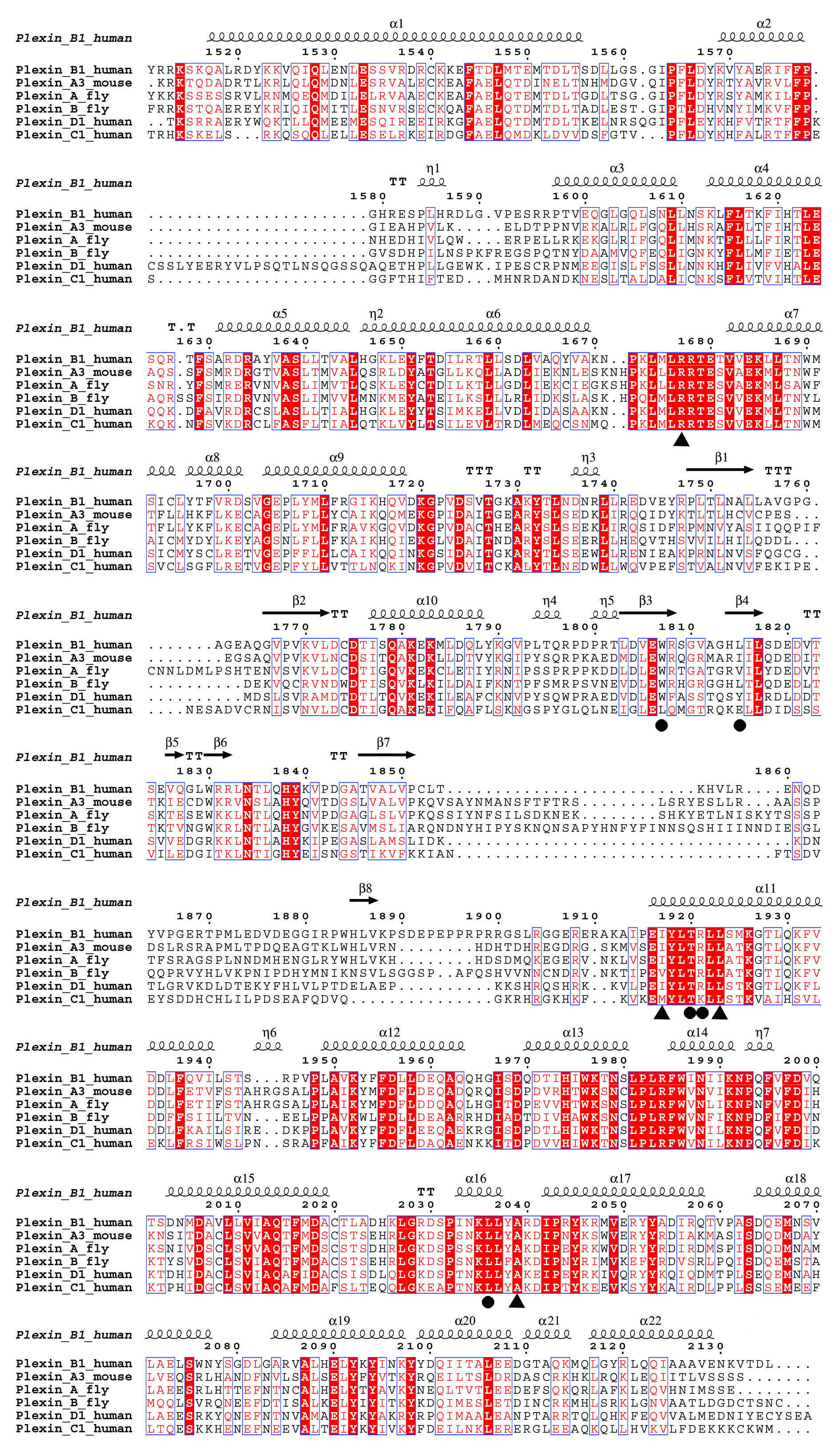

Supplement: Figure S3 — Sequence alignment of the intracellular region of plexins from different classes and organisms. The plexin sequences were aligned using MULTALIN (bioinfo.genotoul.fr/multalin/multalin.html) and formatted with ESPRIPT (espript.ibcp.fr/ESPript/ESPript/). Numbering corresponds to the full-length human Plexin-B1. Secondary structure elements are shown for human Plexin-B1. Residues that were mutated and studied in SPR as well as in the cellular collapse assays are marked with a dot; those that were only studied in the cellular assay are marked with a triangle. (TIF) [file pbio.1001134.s003.tif]

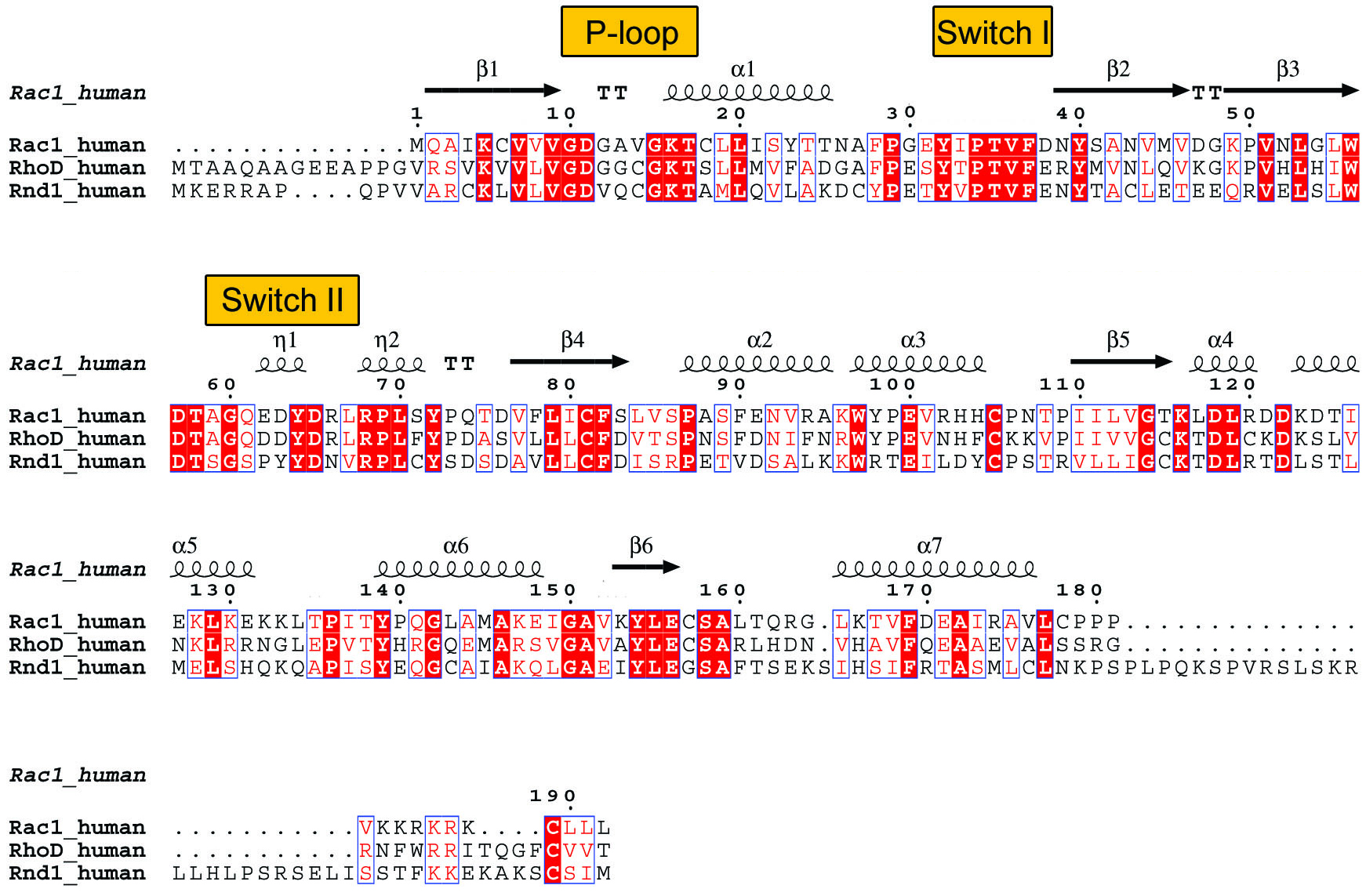

Supplement: Figure S4 — Sequence alignment of the human RhoGTPases Rac1, Rnd1, and RhoD. The alignment is prepared as described in Figure S2. Numbering corresponds to human Rac1. Secondary structure elements are shown for human Rac1. The three regions characteristic for small GTPases and their activation state, the P-loop, switch I, and switch II, are marked by yellow boxes. (TIF) [file pbio.1001134.s004.tif]

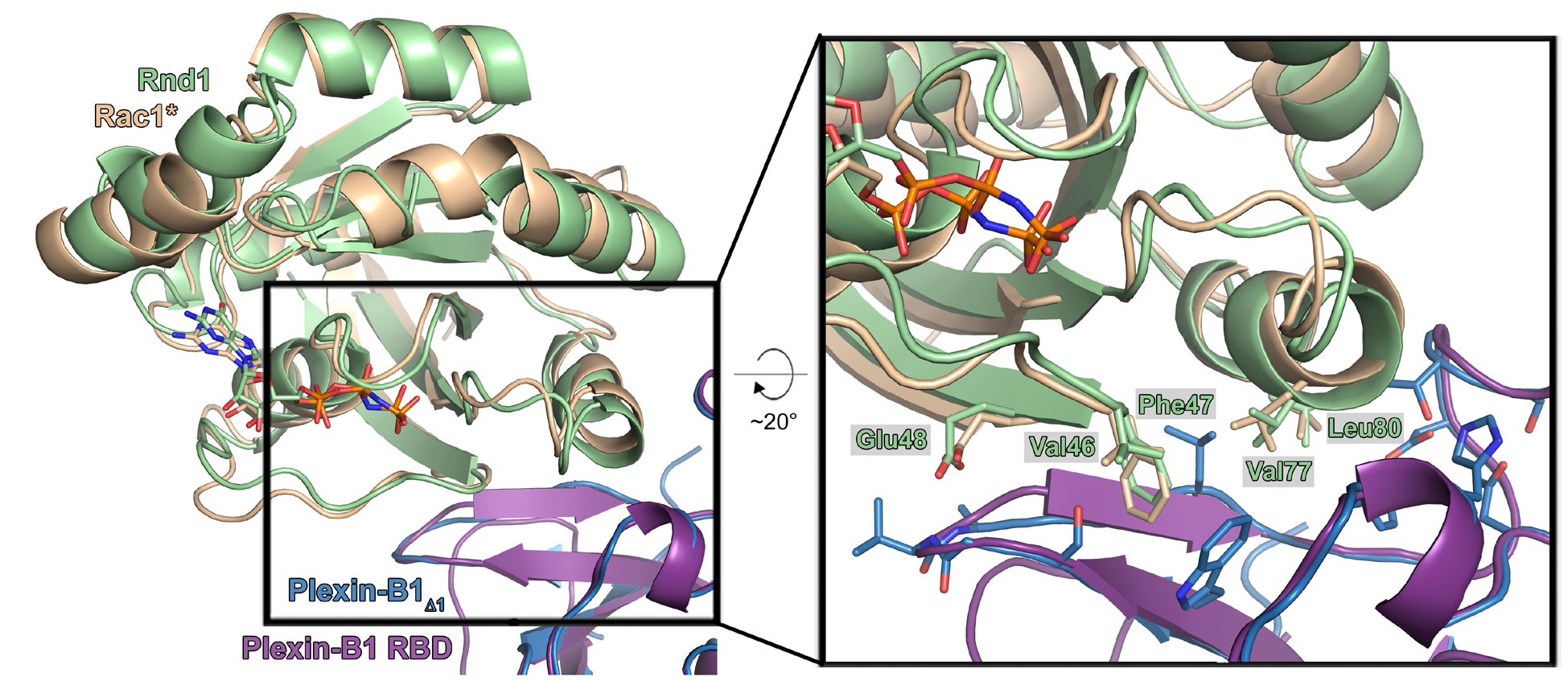

Supplement: Figure S5 — Superposition of the Plexin-B1 RBD-Rnd1 complex onto the Plexin-B1Δ1-Rac1* structure. Colour coding is as in Figure 1b. Rnd1 is in pale green and the Plexin-B1 RBD of the Plexin-B1 RBD-Rnd1 complex in purple. Coordinates for the Plexin-B1 RBD-Rnd1 complex can be found under PDB ID: 2REX. The complexes were aligned onto the plexin molecules using SHP. The orientation is similar to Figure 1b, right panel, with Rnd1 residues labelled in pale green. (TIF) [file pbio.1001134.s005.tif]

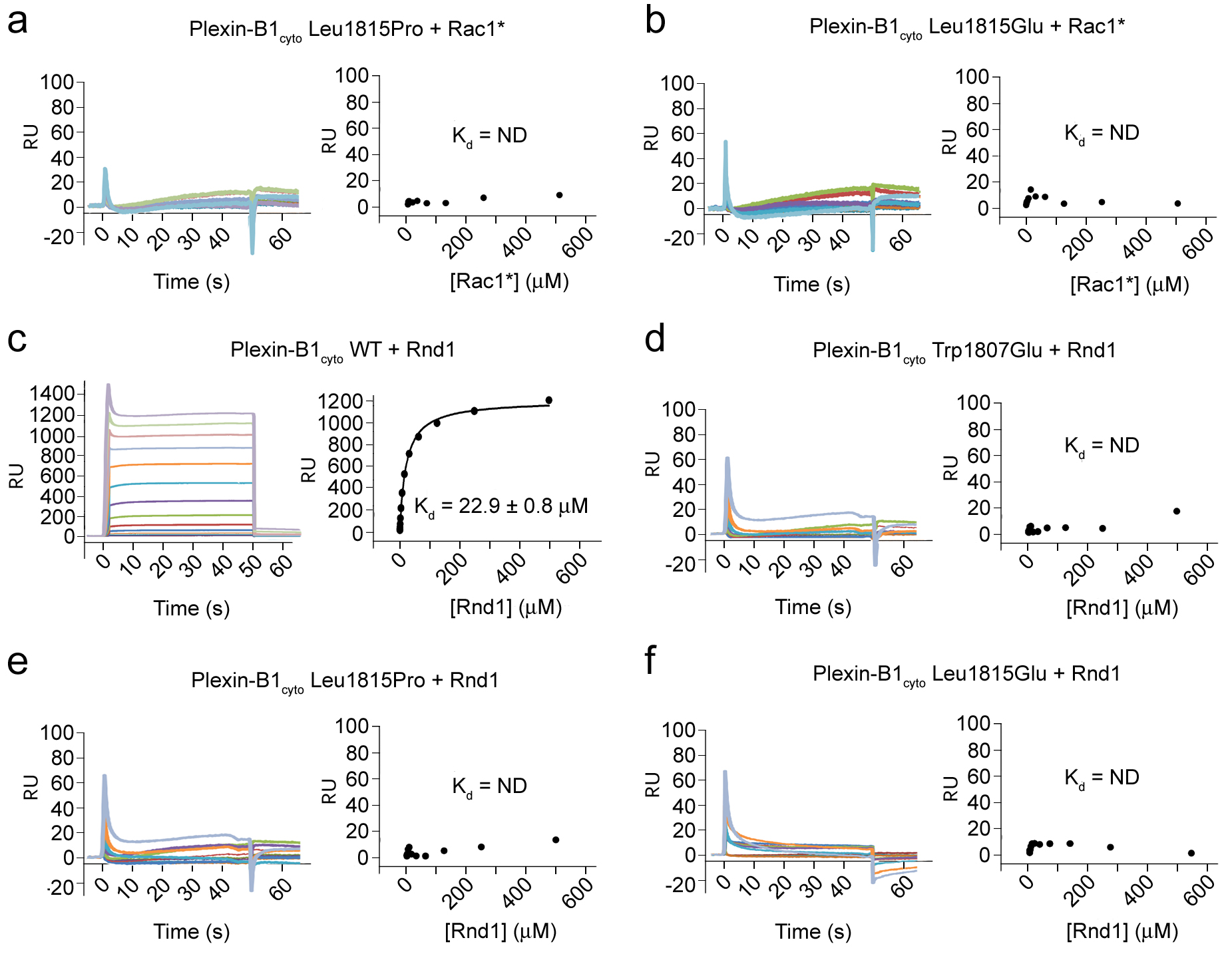

Supplement: Figure S6 — Binding of Rac1* and Rnd1 to site A mutants of Plexin-B1. Left, representative sets of experimental sensorgrams from typical equilibrium-based binding experiments, with reference subtraction. Different concentrations of the respective RhoGTPase were injected over surfaces coupled with the plexin constructs. For all injections, the experimental traces reached equilibrium and returned to baseline after the injection. Right, plot of the equilibrium binding response (response units (RU)) against RhoGTPase concentration ranging from 120 nM to 500 µM. Within one experiment each concentration was measured twice. All experiments were performed in duplicate. Best-fit binding curves corresponding with a 1∶1 binding model are shown as lines. Binding constants (Kd) are given as mean with the error representing the standard error of the mean. WT, wild-type; ND, not determinable. (a) Plexin-B1cyto Leu1815Pro+Rac1*, (b) Plexin-B1cyto Leu1815Glu+Rac1*, (c) Plexin-B1cyto WT+Rnd1, (d) Plexin-B1cyto Trp1815Glu+Rnd1, (e) Plexin-B1cyto Leu1815Pro+Rnd1, and (f) Plexin-B1cyto Leu1815Glu+Rnd1. (TIF) [file pbio.1001134.s006.tif]

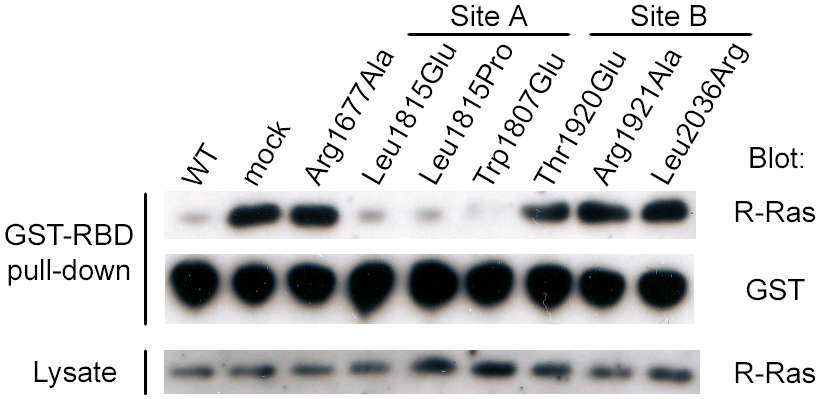

Supplement: Figure S7 — Mutations in site B but not site A abolish Plexin-B1 RasGAP activity. COS-7 cells transfected with full-length Plexin-B1 and its mutants were stimulated with SEMA4Decto for 10 min. The cell lysates were incubated with GST-fused Ras-binding domain of Raf-1 and bound R-Ras and total cell lysates were detected by immunoblotting. The results shown are representative of two independent experiments that yielded similar results. WT, wild-type; mock, chicken receptor protein tyrosine phosphatase σ Ig1-2. (TIF) [file pbio.1001134.s007.tif]

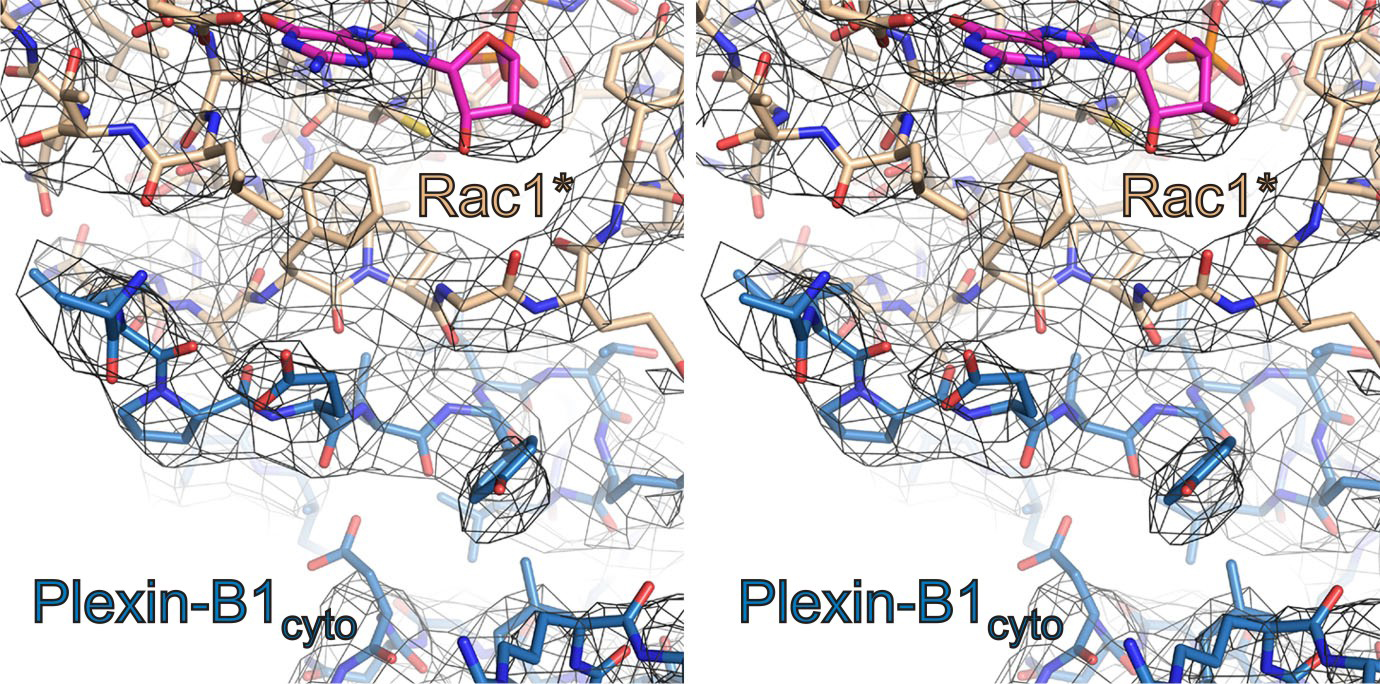

Supplement: Figure S8 — Stereoview of the electron density of the Plexin-B1cyto-Rac1* site B interface. The orientation is similar to Figure 2b, third panel. The density represents a 4.4 Å SigmaA-weighted 2Fobs-Fcalc map contoured at 1.0σ. (TIF) [file pbio.1001134.s008.tif]

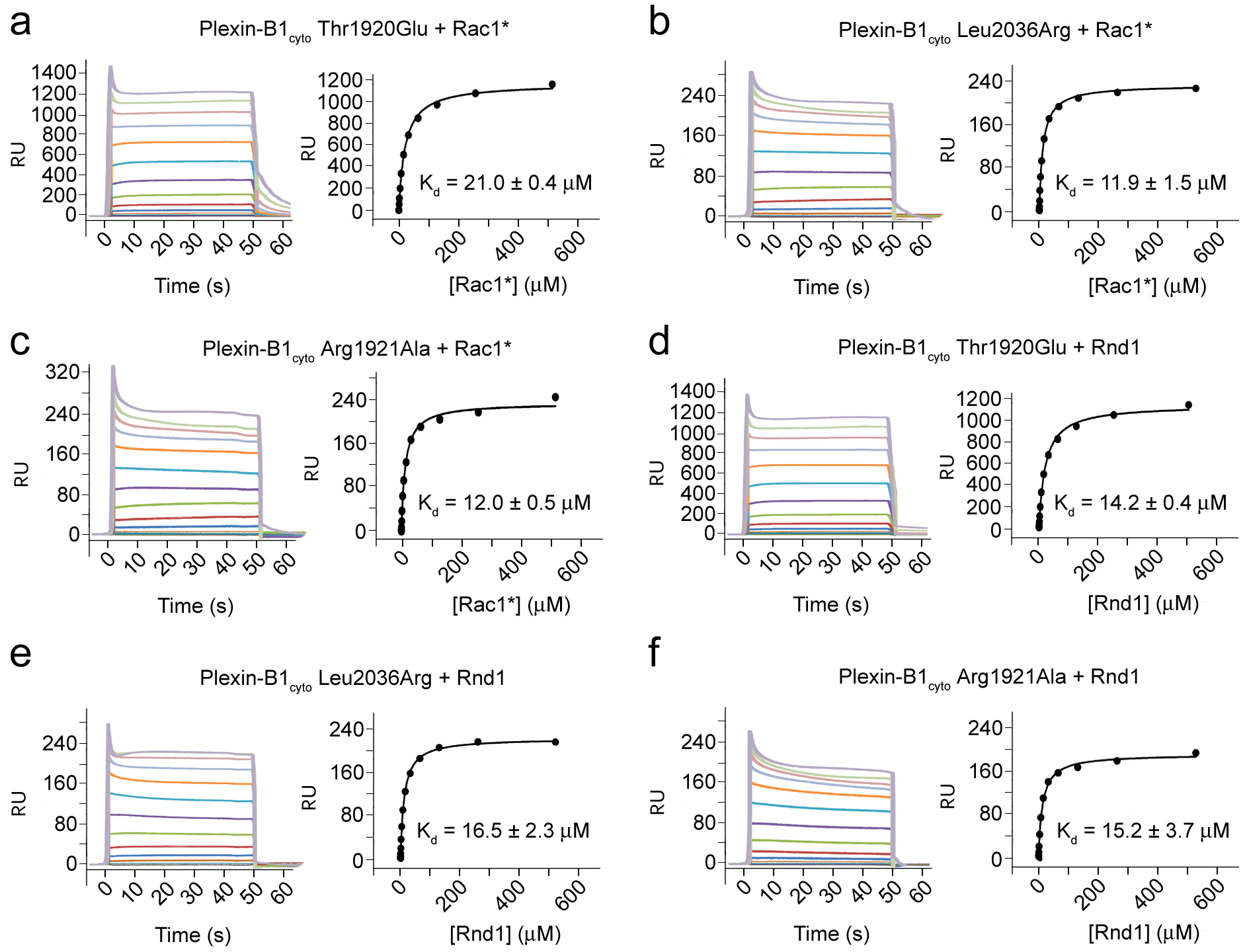

Supplement: Figure S9 — Binding of Rac1* and Rnd1 to site B mutants of Plexin-B1. Data are presented as in Figure S5. (a) Plexin-B1cyto Thr1920Glu+Rac1*, (b) Plexin-B1cyto Leu2036Arg+Rac1*, (c) Plexin-B1cyto Arg1921Ala+Rac1*, (d) Plexin-B1cyto Thr1920Glu+Rnd1, (e) Plexin-B1cyto Leu2036Arg+Rnd1, and (f) Plexin-B1cyto Arg1921Ala+Rnd1. (TIF) [file pbio.1001134.s009.tif]

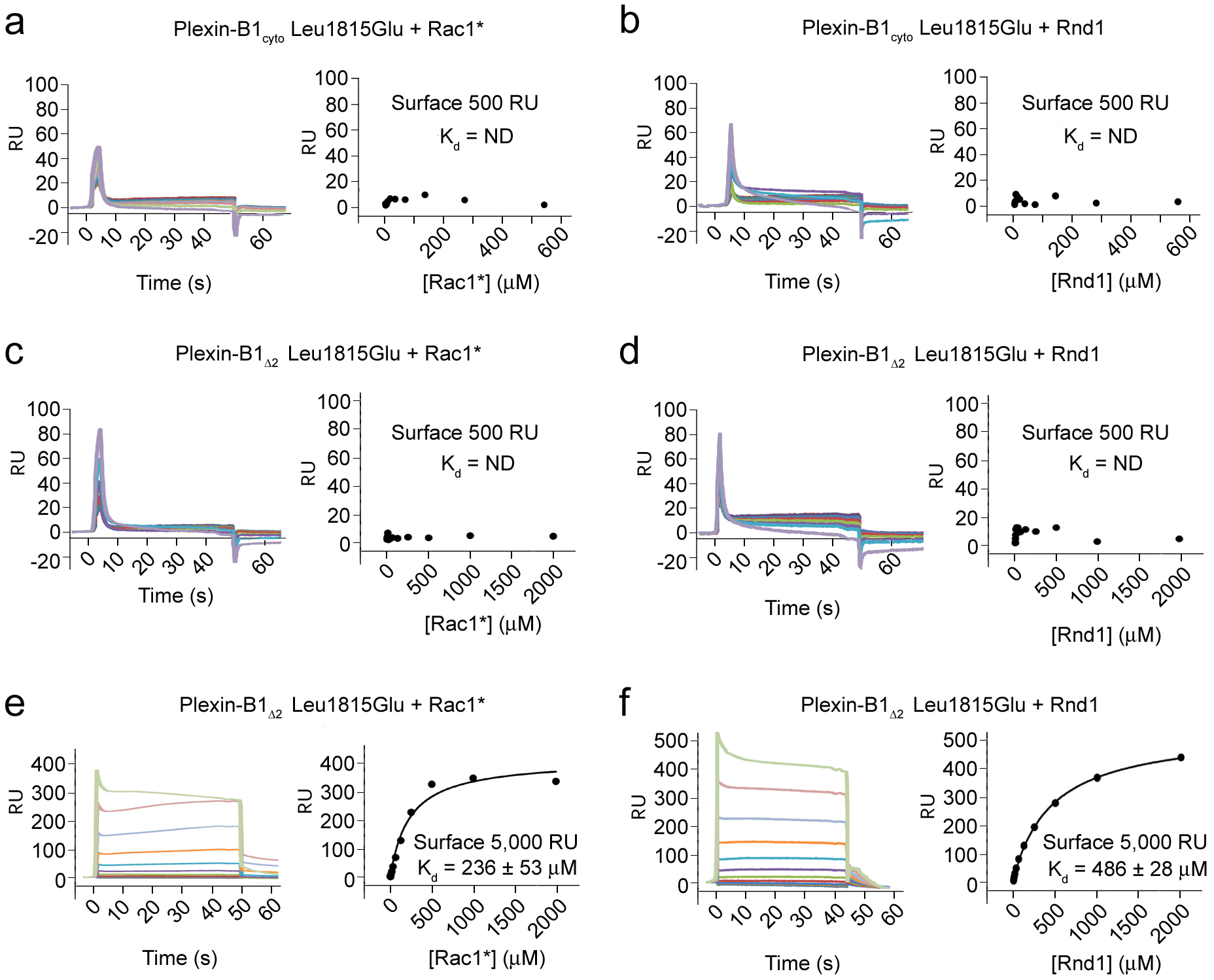

Supplement: Figure S10 — Binding of Rac1* and Rnd1 to Plexin-B1cyto and Plexin-B1Δ2. Data are presented as in Figure S5. (a) Plexin-B1cyto Leu1815Glu+Rac1*, 500 RU loaded on the chip, (b) Plexin-B1cyto Leu1815Glu+Rnd1, 500 RU loaded on the chip, (c) Plexin-B1Δ2 Leu1815Glu+Rac1*, 500 RU loaded on the chip, (d) Plexin-B1Δ2 Leu1815Glu+Rnd1, 500 RU loaded on the chip, (e) Plexin-B1Δ2 Leu1815Glu+Rac1*, 5,000 RU loaded on the chip, and (f) Plexin-B1Δ2 Leu1815Glu+Rnd1, 5,000 RU loaded on the chip. Data for binding of Rac1* or Rnd1 to Plexin-B1cyto Leu1815Glu with 5,000 RU loaded on the chip can be found in Figure S5b and S5f, respectively. (TIF) [file pbio.1001134.s010.tif]

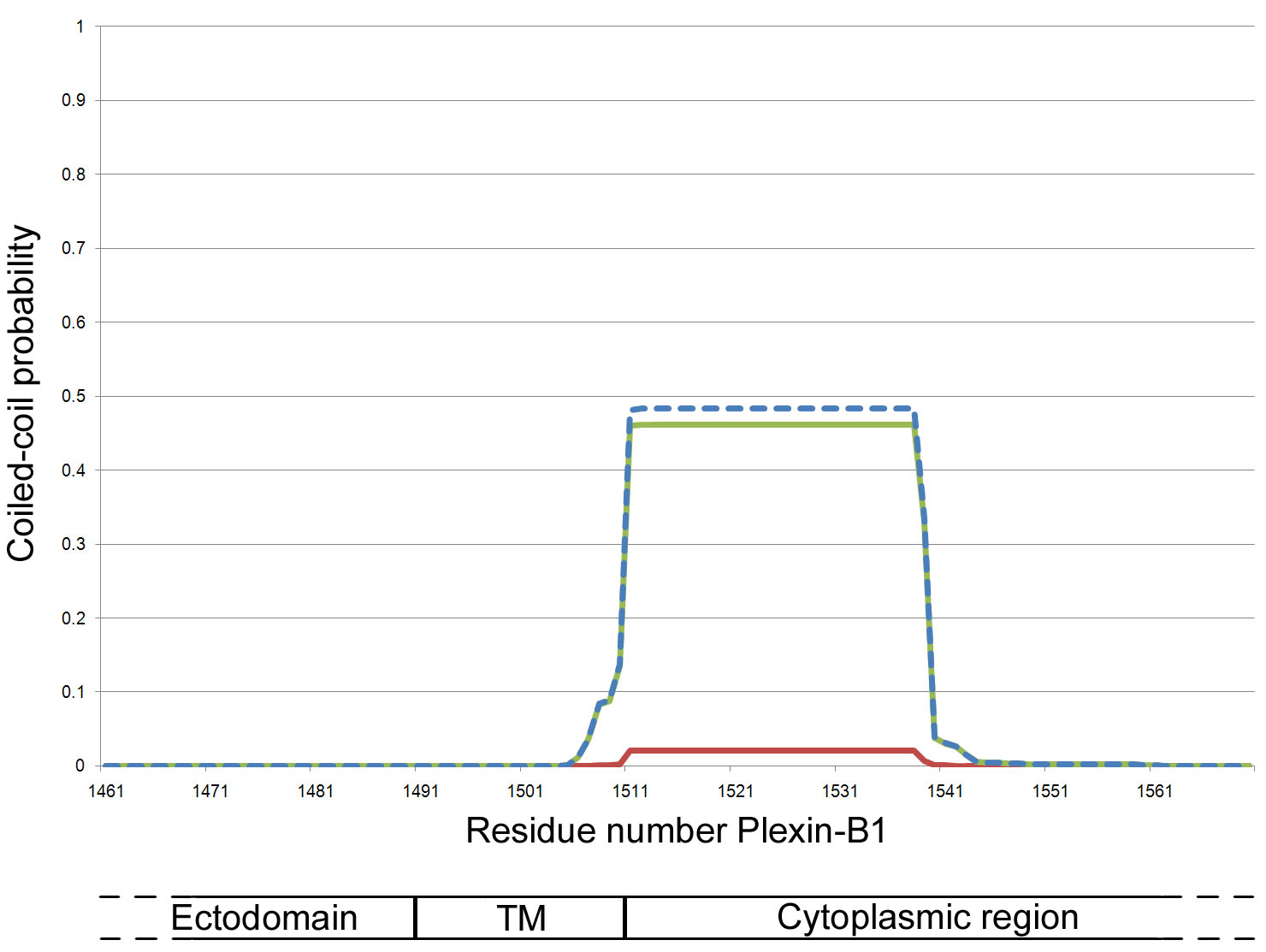

Supplement: Figure S11 — The juxtamembrane helix is predicted to form a trimeric coiled-coil. Coiled-coil probabilities were calculated for human Plexin-B1 using MultiCoil (http://groups.csail.mit.edu/cb/multicoil/cgi-bin/multicoil.cgi) and plotted against residue number. Overall probabilities are shown in dashed blue, trimeric coiled-coil probabilities in green, and dimeric coiled-coil probabilities in red. The domain organization corresponding to the residue numbers is shown under the graph. (TIF) [file pbio.1001134.s011.tif]
